# Effect of a two-phase tobacco control regulation on incidence from ischemic stroke and hemorrhagic stroke, Shenzhen, China, 2007–2016

**DOI:** 10.18332/tid/168123

**Published:** 2023-08-01

**Authors:** Yulin Shi, Ji Peng, Liqun Liu, Zhiguang Zhao, Jingfan Xiong, Xia Wan

**Affiliations:** 1 Department of Epidemiology and Biostatistics, Institute of Basic Medical Sciences Chinese Academy of Medical Sciences, School of Basic Medicine Peking Union Medical College, Beijing, China; 2 Shenzhen Center for Disease Control and Prevention, Shenzhen, China

**Keywords:** smoke-free legislation, second-hand smoke, tobacco, stroke, interrupted time series

## Abstract

**INTRODUCTION:**

The Shenzhen government is widely considered to be most efficiently implementing smoke-free legislation in China. We evaluated and compared the impact of Shenzhen’s two-phase smoke-free regulation on the incidence rates for ischemic and hemorrhagic stroke.

**METHODS:**

An interrupted time series design was used to capture immediate and annual incidence changes from 2007 to 2016 for both ischemic and hemorrhagic stroke due to two-phase smoke-free regulation in Shenzhen, China, by using a generalized additive model. The first phase, implemented on 9 March 2010, required five main public places to be smoke-free. In the second phase, the comprehensive law was expanded to the whole city on 1 March 2014.

**RESULTS:**

The regulation implementation during phase I was associated with a strong immediate decline in the incidence rate of ischemic stroke (-14.2%, 95% CI: -19.6 – -8.4) and hemorrhagic stroke (-10.1%, 95% CI: -18.2 – -1.2), but without showing the annual changes (p>0.05). Following the implementation of the comprehensive law, the gradual annual effect showed a significant change in ischemic stroke, with a 6.3% (95% CI: 8.9 – -3.6) reduction. Neither the immediate nor gradual decreases in hemorrhagic stroke incidences associated with the comprehensive regulation were statistically significant during phase II (p>0.05). Subgroup analyses indicate that a much larger health effect of the regulation during phase I was greater among those aged ≥65 years than among those aged 35–64 years.

**CONCLUSIONS:**

Shenzhen’s two-phase smoke-free regulation was well implemented. Even though the regulation did not extend to the whole city, the immediate health benefits on the incidence rates of ischemic stroke and hemorrhagic stroke could be seen. However, the health benefits brought by the implementation of comprehensive smoke-free legislation were attenuated by previous smoke-free regulations in five main public places, which were more evident in hemorrhagic stroke.

## INTRODUCTION

Implementing comprehensive smoke-free laws could create 100% smoke-free environments, which is the proven way to adequately protect people’s health from the harmful effects of secondhand smoke (SHS)^1^. In addition, scientific evidence has firmly established that an immediate reduction in cardiovascular diseases and respiratory problems would be gained by the comprehensive smoke-free policy’s implementation^2,3^. The World Health Organization (WHO) report of 2021 showed that 67 countries (34%) obtained a Grade I for one of the six MPOWER strategies: Protecting people from tobacco smoke (P), where the smoking bans are at best-practice level, including 1.8 billion people (a quarter of the world’s population) covered by the best-practice smoke-free laws worldwide^4^. In China, smoke-free momentum continues to grow at the subnational level^5^. At present, more than 20 cities have taken promising steps to enact laws or regulations meeting the WHO FCTC requirements^6^.

Shenzhen, as the national economic center and a science and technology innovation center, has been born in the country’s epic reform and opening-up drive and is prospering for its innovation. In 2010, Shenzhen was one of seven cities enrolled in the Bloomberg Initiative project, supported by a joint partnership between the Chinese Center for Disease Control and Prevention (CDC) and the International Union against Tuberculosis and Lung Disease^7^. Under this project support, 100% smoke-free regulation was first implemented in five types of important public places, including hospitals, schools, government agencies, public transportation, and places of public transportation^8^. Four years later, in March 2014, Shenzhen enacted the landmark Shenzhen Special Economic Zone Control Smoking Regulations^9^. It adopted a series of stronger tobacco control policies, including a smoking ban in indoor workplaces and most public places, a prohibition on all advertisement, promotion, and sponsorship of tobacco, and system support for various tobacco control publicity and education on the harms of tobacco use.

The interrupted time series (ITS) design is well-suited to address some unique characteristics of the interventions being studied, including single intervention times and varying durations of intervention roll-out^10,11^. Thus, in this study, the smoke-free regulation in 2010 and the comprehensive law in 2014 were considered as two intervention time points. We aimed to assess the impact of the two-phase tobacco control regulation on the incidence rate for two subtypes of stroke, ischemic and hemorrhagic.

## METHODS

### Data

The incidence data for subtypes of stroke was obtained from the Shenzhen Stroke Registry System, which was established in December 2002. This system currently covers all nine districts in Shenzhen, involving more than 12 million residents, and is responsible for collecting incident stroke reports from 43 secondary and tertiary hospitals. The quality of stroke diagnosis data is assessed and checked by the Shenzhen Center for Chronic Disease Control. The case information collected by the system mainly includes dates of admission, principal diagnosis, sex, date of birth, and household type. The incident stroke was defined as the principal diagnosis for the first stroke event during the study period and the recurrence after 28 days, which was also considered a new stroke case^12^. Based on the International Classification of Diseases, Tenth Version (ICD-10), two significant subtypes of stroke were selected for analysis: ischemic stroke (I63) and hemorrhagic stroke (I60–I62).

The daily meteorological data on the average temperature (°C) and relative humidity (%) were obtained from the Shenzhen Meteorological Bureau. The daily fine particulate matter (PM2.5, particles with an aerodynamic diameter of ≤2.5 μm) concentrations (μg/m^3^) from 2015 to 2016 were obtained from the Shenzhen Environmental Monitoring Center Station. PM2.5 data from 2007 to 2014 were obtained from a near real-time air pollutant concentration database (Tracking Air Pollution in China, TAP, http://tapdata.org.cn/?page_id=523&lang=en), because the local Environmental Monitoring Center Station did not monitor PM2.5 before 2015. This approach to tracking air pollution has been described elsewhere^13^. Single-year population estimates by sex and age were taken from the Shenzhen Bureau of Statistics. Since there was a lack of meteorological data before 2007, the sample was restricted to the resident population aged ≥35 years, between 1 January 2007 and 31 December 2016.

### Setting

The measures for tobacco control in Shenzhen were divided into two phases during the study period. The first phase (phase I), implemented on 9 March 2010, required to be smoke-free in five main public places, including hospitals, schools, government agencies, public transportation and places of public transportation^8^. In the second phase (phase II), a comprehensive law expanded to the whole city was introduced on 1 March 2014^9^.

### Statistical analysis

We combined the number of diagnosed cases with resident population data in Shenzhen to calculate the annual crude incidence rates. Incidence rates were age-adjusted to the Shenzhen population, using the demographic composition in 2017 in Shenzhen. They were stratified by sex and age (35–49, 50–64 and ≥65 years).

A generalized additive model (GAM) developed from an ITS design was used to test the immediate and gradual changes in incidence rates after the two-phase tobacco control regulation. A negative binomial was used in GAM due to the over-dispersion existing in this study. All variables in the model were established as a time series in weeks. The response variable was the crude number of weekly events for two stroke subtypes. The historical trend and seasonal effect in the mode were adjusted using a linear predictor and a Fourier series of sine and cosine terms, respectively. To capture changes of immediate effect of the two-phase of the tobacco control regulation, two indicator variables (X1 and X2) were used to define a smoke-free regulation in five types of important public places (phase I) and a comprehensive law (phase II). To test changes of gradual effect (slope of the secular trend) in phase I and phase II, two interaction terms (X1×T1 and X2×T2) between indicator variables and weeks represented the changes in the slope of weekly incidence rate for phase I versus the period before phase I, and the changes in the slope for phase II versus phase I, respectively. The annual resident population in Shenzhen was included as an offset variable with a fixed coefficient of 1 in each model. In addition, four confounding factors were considered in the model, including number of weekly public holidays, weekly mean temperature, relative humidity and PM2.5. Similar to other studies, a series of separate analyses were conducted in all subgroups (35–64 years, ≥65 years, male, and female).

The immediate changes and annual gradual changes in incidence rates for phase I were quantified as 100[exp(β_2_)-1] and 100[exp(52.1775 β_4_)-1], respectively, and for phase II were 100[exp(β_3_)-1] and 100[exp(52.1775 β_5_)-1] accordingly. Differences in regression coefficients between subgroups were compared with the Chow test. All tests were two tailed, and p<0.05 was considered statistically significant. The data were analyzed using the GAM function in R V.4.1.0.

## RESULTS

### Basic information

Between January 2007 and December 2016, 141237 cases of incident stroke were identified among the resident population aged ≥35 years in Shenzhen, including 110830 cases of incident (78.5%) ischemic stroke and 30407 cases of incident (21.5%) hemorrhagic stroke. The annual incidence rates were 319.1 and 87.5 per 100000 people for ischemic and hemorrhagic stroke, respectively. Overall, an increase year by year in the age-standardized incidence rates for ischemic stroke was observed during the study period. A similar trend was also observed in hemorrhagic stroke, except in 2015 (Table 1). The male incidence rates were higher than those of females for both ischemic stroke (358.9 vs 300.2, per 100000) and hemorrhagic stroke (102.5 vs 71.0, per 100000), respectively. The incidence rates of both ischemic and hemorrhagic stroke increased rapidly with age group, and the differences between the three age groups were statistically significant (Table 2).

**Table 1:** Annual incidence rate* of major subtypes of stroke, Shenzhen, 2007–2016

*Year*	*Ischemic stroke*			*Hemorrhagic stroke*		
	*Number of cases n*	*Crude annual rate (95% CI)*	*Age-standardized rate (95% CI)*	*Number of cases n*	*Crude annual rate (95% CI)*	*Age-standardized rate (95 % CI)*
2007	5336	174.9 (170.2–179.6)	158.4 (153.9–162.9)	1823	59.8 (57.0–62.5)	56.5 (53.8–59.2)
2008	7026	221.5 (216.3–226.6)	199.6 (194.7–204.5)	2287	72.1 (69.1–75.0)	68.4 (65.5–71.3)
2009	8026	242.8 (237.5–248.1)	218.9 (213.8–223.9)	2414	73.0 (70.1–75.9)	69.1 (66.3–71.9)
2010	8420	244.5 (239.3–249.7)	220.8 (215.8–225.8)	2523	73.3 (70.4–76.1)	69.4 (66.6–72.2)
2011	9077	261.3 (255.9–266.7)	237.1 (232.0–242.2)	2701	77.8 (74.8–80.7)	73.9 (71.0–76.7)
2012	10891	311.1 (305.1–317.0)	281.8 (276.3–287.4)	3084	88.1 (85.0–91.2)	83.6 (80.6–86.6)
2013	12508	355.2 (349.0–361.4)	321.0 (315.1–326.9)	3551	100.8 (97.5–104.2)	95.2 (92.0–98.4)
2014	14693	412.5 (405.8–419.1)	372.8 (366.5–379.2)	3716	104.3 (101.0–107.8)	98.7 (95.4–102.0)
2015	16283	432.8 (426.2–439.4)	391.4 (385.1–397.7)	3784	100.6 (97.4–103.8)	95.5 (92.4–98.6)
2016	18570	471.5 (464.8–478.3)	427.2 (420.8–433.6)	4524	114.9 (111.5–118.2)	108.7 (105.4–112.0)
**Total**	110830	319.1 (323.2–325.0)	-	30407	87.5 (84.4–90.6)	-

* Annual incidence rate per 100000.

**Table 2:** Annual incidence rate* of major subtypes of stroke by age and sex, Shenzhen, 2007–2016

*Subgroups*	*Ischemic stroke*			*Hemorrhagic stroke*		
	*Number of cases n*	*Proportion %*	*Average incidence (95% CI)*	*Number of cases n*	*Proportion %*	*Average incidence (95 % CI)*
**Age** (years)						
35–49	17037	15.4	63.3 (60.3–66.3)	10745	35.3	39.9 (37.5–42.3)
50–64	36493	32.9	615.1 (595.2–635.0)	11225	36.9	189.2 (178.2–200.3)
≥65	57300	51.7	3057.3 (2979.3–3135.2)	8437	27.8	450.2 (419.9–480.5)
**Sex**						
Male	65780	59.4	358.9 (350.5–367.3)	19396	63.8	102.5 (98.0–107.0)
Female	45050	40.6	300.2 (291.7–308.7)	11011	36.2	71.0 (66.8–75.1)

* Annual incidence rate per 100000.

### Incidence change before and after the two-phase of tobacco control policy

The segmented negative binomial model described the incidence changes of two subtypes of stroke impacted by the two-phase tobacco control regulation. In terms of ischemic stroke, during phase I, the analysis of the immediate effect showed a significant decrease in incidence rate after the implementation of smoke-free regulation, with a 14.2% (95% CI: -19.6 – -8.4) reduction (Table 3). This decrease was higher among those aged ≥65 years (-19.8%; 95% CI: -25.8 – -13.4) than among those aged 35–64 years (-7.4%; 95% CI: -13.7 – -0.7). The immediate decreases were observed in both males (-13.1%; 95% CI: -19.2 – -6.5) and females (-15.8%; 95% CI: -22.0 – -9.0) as well, with the latter showing a slightly larger decrease. However, the gradual effects were not observed in ischemic stroke (p>0.05) from 2010 to 2014. During phase II, there was no immediate effect of the comprehensive law on the incidence rate of ischemic stroke (p>0.05). However, there was a 6.3% (95% CI: -8.9 – -3.6) incidence decrease per year following the comprehensive law. Similarly, the decreasing trend in the annual incidence was also more pronounced in the higher age groups, with a reduction of 7.8% (95% CI: -10.9 – -4.6). Annual changes were also observed in both men (-5.9%; 95% CI: -8.8 – -2.9) and women (-6.9%; 95% CI: -10.0 – -3.7).

**Table 3:** Multivariate analysis* overall, and by sex and age, after the comprehensive regulation of 2010 and the comprehensive law of 2014, Shenzhen, 2007–2016

*Stroke type*	*Phase 1*				*Phase 2*			
	*Immediate effect (95% CI)*	*p*	*Gradual effect per annum (95% CI)*	*p*	*Immediate effect (95% CI)*	*p*	*Gradual effect per annum (95 % CI)*	*p*
**Ischemic**								
**All**	-14.2 (-19.6 – -8.4)		0.1 (-3.2–3.6)		2.5 (-3.0–8.3)		-6.3 (-8.9 – -3.6)	
**Age** (years)								
35–64	-7.4 (-13.7 – -0.7)	<0.01	0.3 (-3.3–4.0)	0.34	4.6 (-1.2–10.8)	0.46	-4.7 (-7.4 – -1.8)	0.07
≥65	-19.8 (-25.8 – -13.4)		-0.2 (-4.1–3.9)		0.7 (-5.8–7.5)		-7.8 (-10.9 – -4.6)	
**Sex**								
Male	-13.1 (-19.2 – -6.5)	<0.01	0.6 (-3.1–4.5)	0.60	2.0 (-3.9–8.4)	0.94	-5.9 (-8.8 – -2.9)	0.69
Female	-15.8 (-22.0 – -9.0)		-0.5 (-4.4–3.6)		3.1 (-3.5–10.1)		-6.9 (-10.0 – -3.7)	
**Hemorrhagic**								
**All**	-10.1 (-18.2 – -1.2)		4.8 (0.0–9.9)		-5.6 (-13.3–2.9)		-4.9 (-0.6–9.0)	
**Age** (years)								
35–64	-5.5 (-15.5–5.0)	<0.01	5.2 (-0.3–11.0)	0.53	-4.3 (-13.1–5.4)	0.77	-4.6 (-9.2–0.3)	0.78
≥65	-21.4 (-31.0 – -10.4)		4.0 (-2.6–11.0)		-9.9 (-20.1–1.6)		-5.9 (-11.6–0.1)	
**Sex**								
Male	-9.0 (-18.2–1.3)	<0.01	4.9 (-0.6–10.8)	0.74	-4.0 (-12.8–5.7)	0.46	-4.1 (-8.7–0.8)	0.54
Female	-11.9 (-22.0 – -0.5)		4.9 (-1.3–11.6)		-9.1 (-18.9–1.9)		-6.5 (-11.9 – -0.8)	

* Adjusted for time trend, population, seasonality, holiday, temperature, relative humidity and PM2.5.

**Figure 1:**
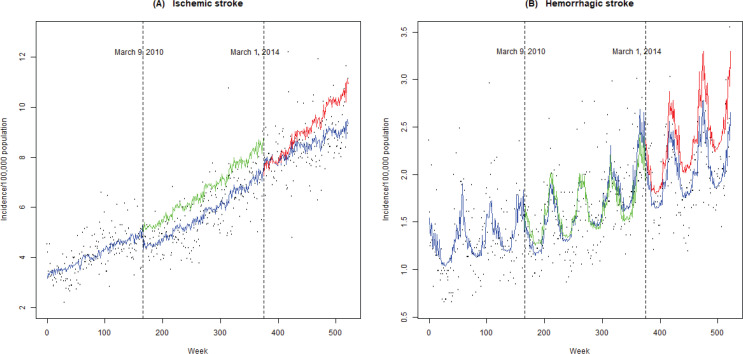
Observed and predicted weekly ischemic and hemorrhagic stroke incidence rates (per 100000) in Shenzhen (2007–2016). Color coding: blue solid line, observed incidence rates; green solid line, predicted incidence rates if no smoke-free regulation; red solid line, predicted incidence rates if no comprehensive smoke-free law; dashed line, enforcement date of both regulation and comprehensive policy

In terms of the hemorrhagic stroke incidence, similar to the trend on ischemic stroke during phase I, 10.1% (95% CI: -18.2 – -1.2) immediate reduction was also observed, but still without the gradual effect. However, the immediate decrease was significant only for females (-11.9%; 95% CI: -22.0 – -0.55) and those aged ≥65 years (-21.4%; 95% CI: -31.0 – -10.4). It is noted that immediate and gradual reductions in the hemorrhagic stroke incidence during phase II were observed, but without statistical significance. The weekly variation followed a seasonal pattern with higher incidence rates in winter and lower rates during summer, which was particularly evident in hemorrhagic stroke (Figure 1).

## DISCUSSION

In this study, we analyzed the two-phase smoke-free regulation on the incidence rates for ischemic and hemorrhagic stroke. Previously, when conducting the smoke-free legislation performance evaluation, acute myocardial infarction was the preferred disease. Analyzing the stroke by different subtypes is not quite common, especially under the two-phase smoke-free regulation circumstances. According to the Surgeon General’s Report from 2006 to 2014, the evidence for the causal relationship between increased risk of stroke and exposure to SHS changed from insufficient to sufficient^14^. Therefore, this study adds more evidence for this inference, as well as the smoke-free policy performance assessment.

During phase I, irrespective if it was an ischemic or hemorrhagic stroke, an immediate reduction caused by the regulation in the main five public places was observed, but the gradual change was not seen for both subtypes of stroke. During phase II, only a gradual effect with significance was detected in the ischemic stroke. These changes for the immediate reduction of phase I were consistent with the fast-acting harmful effects of SHS on stroke, such as increasing platelet activation and aggregation and altering endothelial function within 30 minutes, which can be observed even at low-dose environmental tobacco exposure^15-17^. Our above results are in line with the previous research results, such as a meta-analysis^18^, and studies in Beijing^16^, Arizona^19^, and Florida^20^, which also found significant immediate effects on stroke following a smoke-free law. Since this smoke-free regulation during Phase I was not extended to the whole city, the health effects were limited, and gradual changes were not observed in two subtypes of stroke. However, a 100% smoke-free environment created in five types of public places also protected non-smokers from SHS exposure, resulting in an immediate decrease in stroke incidence.

During phase II, immediate decreases on incidence of ischemic and hemorrhagic stroke were not detected. One possible explanation is that the health effects of the comprehensive smoke-free legislation on stroke have shifted to phase I. But our model still captured a gradual change (-6.3%; 95% CI: -8.9 – -3.6) after the comprehensive law on ischemic stroke. Our results represent the difference in gradual effects between ischemic stroke and hemorrhagic stroke at this phase. Gradual change (-6.3%; 95% CI: -8.9 – -3.6) for ischemic stroke was of significance, but not for hemorrhagic stroke (-4.9%; 95% CI: -0.6 – 9.0), which might be explained by a difference in pathogenesis. The Surgeon General Report 2014 mentioned that the relative strength of the association between active smoking and cerebrovascular events was stronger for ischemic stroke than for hemorrhagic stroke, which might suggest the similar mechanism difference between these two subtypes when people are exposed to SHS^14^. Therefore, some findings have emphasized the need to carefully consider ischemic and hemorrhagic stroke as separate entities^21^. The Mackay et al.^22^ study showed there was a dramatic fall in cerebral infarctions after the legislation’s performance for around 20 months in Scotland, but not in other types of stroke, which had similar results as ours. Although in phase II, immediate and gradual reductions in the hemorrhagic stroke incidence was observed, but without statistically significant. We assumed that the short span of time for policy implementation might be one of the reasons. In general, our results suggested that ischemic stroke might be more sensitive to smoke-free legislation compared to hemorrhagic stroke.

The negative binomial regression model developed using a three-segmented ITS allowed us to differentiate the health effects of two interventions of different intensities. It is quite common that some countries and regions often formulate administrative regulations or policies or conduct tobacco control initiatives before the implementation of comprehensive smoke-free legislation. Therefore, when we evaluate the health effect of a comprehensive smoke-free law at the city level, the impact is always muted by those initiatives^23,24^. Our findings also confirmed this. Thus, this lack of significance in immediate or annual changes in disease morbidity cannot be attributed to poor enforcement of the comprehensive smoke-free law because the health effect caused by the smoke-free law might have shifted forward. Overall, we recommend that when the health effect is used to evaluate smoke-free law enforcement, the relative tobacco control initiatives should be considered with the multi-segmented ITS design.

Several studies conducted in North American, European and Asian countries reported that diverse health effects of smoke-free law in different age groups^12,16,18,22,25^. Similar findings were found in our study. We found the association between smoke-free laws and stroke incidence rates was stronger among elderly people. One possible explanation is that, in China, most young people prefer to travel by private transportation, cycling, and walking, which were not protected in the smoke-free regulation during phase I. In contrast, most elderly people prefer to travel by public transportation, where the most concerned areas are. Therefore, the levels of SHS exposure in these public places have dropped significantly after the smoke-free regulation. Our study suggests that immediate and gradual decreases in stroke incidence rates were greater among females than among males, which were compatible with findings from Qingdao, China^12^. This might be explained by the different distribution of smoking prevalence and SHS exposure between genders.

From these results, we can see that even though the regulation did not extend to the whole city, just focusing on the five types of important public places, once it was implemented well, the health benefits could be seen. In addition, the gradual effect on both diseases was seen by the implementation of the comprehensive smoke-free policy in 2014, which means the law in Shenzhen was implemented well. In the WHO report of 2017, Shenzhen was also mentioned as one of the major cities in China that became smoke-free^26^. The Chinese government set a target on tobacco control in the Healthy China 2030 Strategy, which requires that 30% and 80% of the population to be protected by complete smoking bans by 2022 and 2030, respectively^27,28^. However, currently in China, only 0.216 million people (15.3%) are protected by the smoke-free environment, which shows a huge gap from the Healthy China 2030 targets^29^. Therefore, if the smoking prevalence rate reaches the goal of Healthy China 2030, as we expect, one of the most effective ways is to adequately implement the existing subnational comprehensive smoke-free law.

### Strengths and limitations

This study had three strengths over previous research. First, our study was conducted over a longer period of time than most other studies, and hospital admissions were captured through 12 million population databases, allowing for better delineation of trends. Second, this study was the first to compare the health effects of two-phase tobacco control regulations in China. Finally, given that the mechanisms of hemorrhagic stroke and ischemic stroke are different, we assessed the potential health effects on these two subtypes of stroke following the implementation of tobacco control policies, respectively. There are two limitations to our study. First, despite adjusting for highly relevant covariates (time trends, PM2.5, etc.) and seasonality, there were still other factors we were unable to control for (prevalence of hypertension and atrial fibrillation, obesity prevalence, population cholesterol levels, etc.). Also, individual information was unavailable on covariates (smoking status) due to this study’s ecological design. Previous studies that differentiated the effects of the law on smoking and non-smoking populations in terms of cardiovascular diseases found a greater effect in non-smoking populations^30,31^. Thus, if we could distinguish between smokers and non-smokers in our study, more health effects of tobacco control policy would have been observed in non-smokers.

## CONCLUSIONS

By analyzing incidence data from the Shenzhen Stroke Registry System covering about 12 million people over 10 years, we found that the smoke-free regulation in Shenzhen was well implemented and that a health benefit has been gained with regard to stroke, especially for older people. Compared to hemorrhagic stroke, ischemic stroke was more sensitive to the law. The health benefits brought by the implementation of comprehensive smoke-free legislation were attenuated by previous smoke-free regulations in five main public places. Thus, during the smoke-free law enforcement evaluation, the related tobacco control initiatives or campaigns conducted prior to the smoke-free legislation being enacted should be considered.

## Data Availability

These data cannot be shared because of relative regulations from local agency.

## References

[1] World Health Organization (2008). WHO Framework Convention on Tobacco Control: a powerful tool.

[2] Been JV, Nurmatov UB, Cox B, Nawrot TS, van Schayck CP, Sheikh A (2014). Effect of smoke-free legislation on perinatal and child health: a systematic review and meta-analysis. Lancet.

[3] Jones MR, Barnoya J, Stranges S, Losonczy L, Navas-Acien A (2014). Cardiovascular events following smoke-free legislations: an updated systematic review and meta-analysis. Curr Environ Health Rep.

[4] World Health Organization (2021). WHO report on the global tobacco epidemic 2021: addressing new and emerging products.

[5] Song L, Luo X, Yang J (2022). Feasibility and practicability studies of an evaluation index system for implementation of smoke-free laws and regulations of cities in China. Tob Induc Dis.

[6] Lin H, Chang C, Liu Z, Zheng Y (2019). Subnational smoke-free laws in China. Tob Induc Dis.

[7] Yang G (2018). Tobacco Control in China.

[8] Tobacco China (2010). Smoke-free Environment Promotion Project.

[9] People’s Government of Guangdong Provincial (2019). Shenzhen Special Economic Zone Smoking Control Regulations.

[10] Habib N, Steyn PS, Boydell V (2021). The use of segmented regression for evaluation of an interrupted time series study involving complex intervention: the CaPSAI project experience. Health Serv Outcomes Res Methodol.

[11] Bernal JL, Cummins S, Gasparrini A (2017). Interrupted time series regression for the evaluation of public health interventions: a tutorial. Int J Epidemiol.

[12] Xiao H, Qi F, Jia X (2020). Impact of Qingdao’s smoke-free legislation on hospitalizations and mortality from acute myocardial infarction and stroke: an interrupted time-series analysis. Addiction.

[13] Geng G, Xiao Q, Liu S (2021). Tracking air pollution in China: near real-time PM2.5 retrievals from multisource data fusion. Environ Sci Technol.

[14] National Center for Chronic Disease Prevention and Health Promotion (US) Office on Smoking and Health (2014). The Health Consequences of Smoking—50 Years of Progress: A Report of the Surgeon General.

[15] Law MR, Wald NJ (2003). Environmental tobacco smoke and ischemic heart disease. Prog Cardiovasc Dis.

[16] Zheng Y, Wu Y, Wang M (2020). Impact of a comprehensive tobacco control policy package on acute myocardial infarction and stroke hospital admissions in Beijing, China: interrupted time series study. Tob Control.

[17] Puranik R, Celermajer DS (2003). Smoking and endothelial function. Prog Cardiovasc Dis.

[18] Tan CE, Glantz SA (2012). Association between smoke-free legislation and hospitalizations for cardiac, cerebrovascular, and respiratory diseases: a meta-analysis. Circulation.

[19] Herman PM, Walsh ME (2011). Hospital admissions for acute myocardial infarction, angina, stroke, and asthma after implementation of Arizona’s comprehensive statewide smoking ban. Am J Public Health.

[20] Loomis BR, Juster HR (2012). Association of indoor smoke-free air laws with hospital admissions for acute myocardial infarction and stroke in three states. J Environ Public Health.

[21] Chauhan G, Debette S (2016). Genetic risk factors for ischemic and hemorrhagic stroke. Curr Cardiol Rep.

[22] Mackay DF, Haw S, Newby DE (2013). Impact of Scotland’s comprehensive, smoke-free legislation on stroke. PLoS One.

[23] Rodu B, Peiper N, Cole P (2012). Acute myocardial infarction mortality before and after state-wide smoking bans. J Community Health.

[24] Juster HR, Loomis BR, Hinman TM (2007). Declines in hospital admissions for acute myocardial infarction in New York state after implementation of a comprehensive smoking ban. Am J Public Health.

[25] Mayne SL, Widome R, Carroll AJ (2018). Longitudinal associations of smoke-free policies and incident cardiovascular disease: CARDIA Study. Circulation.

[26] World Health Organization (2017). WHO report on the global tobacco epidemic, 2017: monitoring tobacco use and prevention policies.

[27] (2019). The State Council of the People’s Republic of China.

[28] Shi YL, Zhao J, Ai FL (2022). Evaluating the quality of case-control studies involving the association between tobacco exposure and diseases in a Chinese population based on the Newcastle-Ottawa Scale and Post-hoc Power. Biomed Environ Sci.

[29] Caixin Data Visualization Lab Healthy China targets finish rate.

[30] Frazer K, Callinan JE, McHugh J (2016). Legislative smoking bans for reducing harms from secondhand smoke exposure, smoking prevalence and tobacco consumption. Cochrane Database Syst Rev.

[31] Agüero F, Dégano IR, Subirana I (2013). Impact of a partial smoke-free legislation on myocardial infarction incidence, mortality and case-fatality in a population-based registry: the REGICOR Study. PLoS One.

